# Serologic Cross-Reactivity of Human IgM and IgG Antibodies to Five Species of Ebola Virus

**DOI:** 10.1371/journal.pntd.0001175

**Published:** 2011-06-07

**Authors:** Adam MacNeil, Zachary Reed, Pierre E. Rollin

**Affiliations:** Viral Special Pathogens Branch, The Centers for Disease Control and Prevention, Atlanta, Georgia, United States of America; University of Texas Medical Branch at Galveston, United States of America

## Abstract

Five species of Ebola virus (EBOV) have been identified, with nucleotide differences of 30–45% between species. Four of these species have been shown to cause Ebola hemorrhagic fever (EHF) in humans and a fifth species (*Reston ebolavirus*) is capable of causing a similar disease in non-human primates. While examining potential serologic cross-reactivity between EBOV species is important for diagnostic assays as well as putative vaccines, the nature of cross-reactive antibodies following EBOV infection has not been thoroughly characterized. In order to examine cross-reactivity of human serologic responses to EBOV, we developed antigen preparations for all five EBOV species, and compared serologic responses by IgM capture and IgG enzyme-linked immunosorbent assay (ELISA) in groups of convalescent diagnostic sera from outbreaks in Kikwit, Democratic Republic of Congo (n = 24), Gulu, Uganda (n = 20), Bundibugyo, Uganda (n = 33), and the Philippines (n = 18), which represent outbreaks due to four different EBOV species. For groups of samples from Kikwit, Gulu, and Bundibugyo, some limited IgM cross-reactivity was noted between heterologous sera-antigen pairs, however, IgM responses were largely stronger against autologous antigen. In some instances IgG responses were higher to autologous antigen than heterologous antigen, however, in contrast to IgM responses, we observed strong cross-reactive IgG antibody responses to heterologous antigens among all sets of samples. Finally, we examined autologous IgM and IgG antibody levels, relative to time following EHF onset, and observed early peaking and declining IgM antibody levels (by 80 days) and early development and persistence of IgG antibodies among all samples, implying a consistent pattern of antibody kinetics, regardless of EBOV species. Our findings demonstrate limited cross-reactivity of IgM antibodies to EBOV, however, the stronger tendency for cross-reactive IgG antibody responses can largely circumvent limitations in the utility of heterologous antigen for diagnostic assays and may assist in the development of antibody-mediated vaccines to EBOV.

## Author Summary

Ebola virus (EBOV) is a highly pathogenic virus, capable of causing Ebola hemorrhagic fever in humans and non-human primates. Five species of EBOV have been identified. To examine whether infection with one EBOV species results in antibodies that cross-react with other EBOV species, we selected groups of human diagnostic samples from four outbreaks, which were each due to a different EBOV species, and compared IgM and IgG responses by ELISA to each of the five EBOV species. For samples from an individual outbreak, we found limited IgM reactivity to species of EBOV other than the virus species the individual was infected with. In contrast, for all groups of outbreak samples we observed strong cross-reactive IgG antibodies to all EBOV species. Our study demonstrates that IgG antibody responses tend to be more cross-reactive than IgM antibody responses in people infected with EBOV, a finding that has implications for the development of diagnostic assays and vaccines to EBOV.

## Introduction

The genus *Ebolavirus*, family *Filoviridae*, has five identified (including one proposed) viral species [1]. Of these, four viral species, *Zaire ebolavirus* (ZEBOV), *Sudan ebolavirus* (SEBOV), *Côte d'Ivoire ebolavirus* (CIEBOV), and *Bundibugyo ebolavirus* (BEBOV) are known to cause Ebola hemorrhagic fever (EHF) in humans, and in previous large outbreaks due to ZEBOV, SEBOV, and BEBOV, case fatality has ranged from 32 to 90% [2], [3], [4], [5], [6], [7], [8], [9]. A fifth viral species, *Reston ebolavirus* (REBOV), has been shown to cause severe disease in non-human primates [10], [11], [12] and can infect swine [13]. Similarly, evidence of human infections with REBOV have been documented serologically, however, no human disease has been associated with REBOV [13], [14], [15]. Despite the common characteristic of severe pathogenic potential in humans or non-human primates, genomic sequencing indicates relatively high divergence between Ebola viruses, with nucleotide differences ranging from 30–45% between species [16].

The role of antibody response in viral clearance and protective immunity against Ebola viruses in humans is not fully understood, however samples from individuals with acute ZEBOV infection have demonstrated antibodies titers that peak relatively early among those who survive, whereas low or absent antibody titers are commonly present in those with a fatal outcome [17], [18]. Similarly, others have reported the presence of detectable anti-EBOV antibodies in humans during acute EHF (in some instances with concurrent detectable viremia [16], [19], [20]), as well as in asymptomatic individuals shortly after exposure [21], [22], again suggesting that antibody response may be a correlate of protective immunity to EHF.

EHF outbreaks commonly occur in remote locations, and often there is a significant lag between the occurrence of initial illnesses and subsequent diagnostic sample collection. As a result, diagnostic samples are frequently collected from individuals following clearance of viremia, only allowing serologic diagnosis of EHF. Adding to the challenge in EHF diagnosis, is the near geographic overlap of at least three pathogenic EBOV species (ZEBOV, SEBOV, and BEBOV) in central Africa [23], [24]. While we previously have had success in the serologic diagnosis of EBOV infection using heterologous antigen (for instance, BEBOV was initially identified by IgM reactivity to ZEBOV antigen [16]), the overall genetic divergence between EBOV species remains a concern, and previous data has suggested potential differences in serologic reactivity to different EBOV species in humans with EHF [25], [26]. In order to examine the extent of serologic cross-reactivity of EBOV, as well as assess the utility of heterologous viral antigen for diagnosis of EBOV infection, we generated non-recombinant, infectious virus-based antigen preparations for the five known EBOV species, and examined the IgM and IgG responses against all five viruses in human sera collected from previous outbreak responses, associated with ZEBOV, SEBOV, BEBOV, and REBOV.

## Methods

### Ethics Statement

All samples were collected as part of public health diagnostic activities, were pre-existing relative to the start of the study, and were examined as anonymous samples. Ethical review of the study protocol was performed by the CDC Investigational Review Board and study approval was obtained following review, from the CDC Human Research Protection Office.

### Sample selection

Samples for this current study were previously collected as part of EHF outbreak responses, for 24 individuals infected with ZEBOV (Kikwit, Democratic Republic of Congo, 1995 [5]), 20 individuals infected with SEBOV (Gulu, Uganda, 2000 [19]), and 33 individuals infected with BEBOV (Bundibugyo, Uganda, 2007 [9]) (table 1). In addition, we assessed antibody responses in 18 samples that were collected from humans in the Philippines and sent to CDC for confirmatory testing, following the 2008 detection of REBOV in swine [13]. During diagnostic testing at CDC, the Philippines samples were found positive for REBOV-reactive IgG antibodies; the date of onset, or even previous occurrence of illness in individuals from whom these samples were obtained is unknown. While the time of sample collection, relative to disease onset differed between outbreaks (with samples from Gulu tending to be from earlier stages post-infection than samples from Bundibugyo or Kikwit), all samples were from individuals who survived EBOV infection, and diagnostic testing at the time of outbreak response demonstrated the absence of viremia (by PCR or antigen detection ELISA) and the presence of IgG antibodies in each the samples included in this study. Each sample included in this study is from a discrete individual.

**Table 1:** Summary information on study samples.

Outbreak, year	EBOV species responsible for outbreak	Number of samples included in current study	Median (days) time of sample collection, post-symptom onset	Range (days) of samples collected, post-symptom onset
Kitwit, DRC, 1995	ZEBOV	24	73.5	34–116
Gulu, Uganda, 2000	SEBOV	20	18	14–70
Bundibugyo, Uganda, 2007	BEBOV	33	48	33–117
Philippines, 2008	REBOV	18	Unknown	Unknown

### Serology

Antigen preparations for IgM and IgG assays were performed as described previously [17], [27]. Briefly, viral antigens for IgM and IgG ELISA were prepared by viral culture in Vero E6 cells, and harvested when at least 90% of cells had evidence of infection by immunofluorescence assay. Infected cells were processed by lysis of cells and supernatant for slurry antigen preparations (IgM) or by detergent basic buffer extraction of infected cells for lysate antigen preparations (IgG), as described previously [17], [27]. While the approach for antigen preparation does differ in terms of antigen concentration between IgM and IgG assays, the viral antigenic components are similar between both approaches. The decision to use these specific approaches is based on previously optimized protocols, which have been applied in numerous diagnostic settings. Viral antigen preparations were developed for each of the five known EBOV species, using viral isolates the following outbreaks: Kikwit, Democratic Republic of Congo, 1995 (ZEBOV) [5], Gulu, Uganda, 2000 (SEBOV) [19], Bundibugyo, Uganda, 2007 (BEBOV) [9], the Philippines (isolate from swine tissue sample submitted to USDA), 2008 (REBOV) [13], and Tai Forest, Côte d'Ivoire, 1994 (CIEBOV) [6]. Mock-infected control antigens for IgM and IgG assays were prepared in similar manners, respectively, in the absence of virus.

Western blots on viral antigen preparations were performed as described previously [28], individually using hyperimmune mouse ascitic fluid (HMAF) poly-clonal antibodies [29] against ZEBOV, SEBOV, REBOV, and CIEBOV, and rabbit poly-clonal antibodies against ZEBOV, SEBOV, and REBOV, for detection of EBOV proteins; secondary antibodies were goat anti-mouse IgG horseradish peroxidase conjugate and goat anti-rabbit IgG horseradish peroxidase conjugate, respectively.

IgM capture and IgG ELISAs were performed using Clinical Laboratory Improvement Amendments (CLIA) certified protocols that have been used for diagnostic testing of EBOV since 1990 [17], [27]. For IgM assay, we used goat anti-human IgM antibody (1∶500) for antigen capture, slurry antigen preparations (1∶1000), an HMAF poly-clonal antibody mixture, raised against ZEBOV, SEBOV, REBOV, and CIEBOV as detector antibody (1∶2000), and anti-mouse IgG horseradish peroxidase conjugate (1∶8000) and ABTS substrate. For IgG assay, we used lysate antigen preparations (1∶1000) and mouse anti-human IgG horseradish peroxidase conjugate (1∶4000) and ABTS substrate. ELISAs were performed for samples, using both viral antigen and mock-infected antigen, at dilutions of 1∶100, 1∶400, 1∶1600, and 1∶6400. Adjusted optical density (OD) values represent the OD value (at 410 nm) of an individual sample dilution, after subtracting the OD value of mock infected antigen from the viral antigen for that dilution. The adjusted sum OD represents the sum of adjusted OD values of the four dilutions for an individual sample. For diagnostic assessment of antibody responses, individual sample dilutions with an adjusted OD of ≥0.1 (IgM ELISA) or ≥0.20 (IgG ELISA) were considered positive at that respective dilution, and an antibody response was considered positive for a sample if the sample had a positive titer of at least 1∶400 plus an adjusted sum OD of ≥0.45 (IgM ELISA) or ≥0.95 (IgG ELISA). Cut-off values for the adjusted OD and adjusted sum OD for both assays correspond with diagnostic criteria currently used for EHF rule-out testing by CDC, and are based on previous evaluation of the distribution of values from thousands of negative serologic samples.

### Data analysis

For statistical comparison of adjusted sum OD values between autologous (reaction to the same EBOV species that the individual was infected with) and heterologous (reaction to different EBOV species that the individual was infected with) virus antigen preparations, we selected all samples from a single outbreak and performed Wilcoxon rank sum tests, for a non-parametric paired sample comparison of adjusted sum OD values. That is, for an individual outbreak, for each sample we calculated the difference in adjusted sum OD values between autologous antigen and a single heterologous antigen and tested whether the distribution of differences for all samples for that autologous-heterologous antigen pair was different from zero, by Wilcoxon rank sum test.

## Results

**Figure 1:**
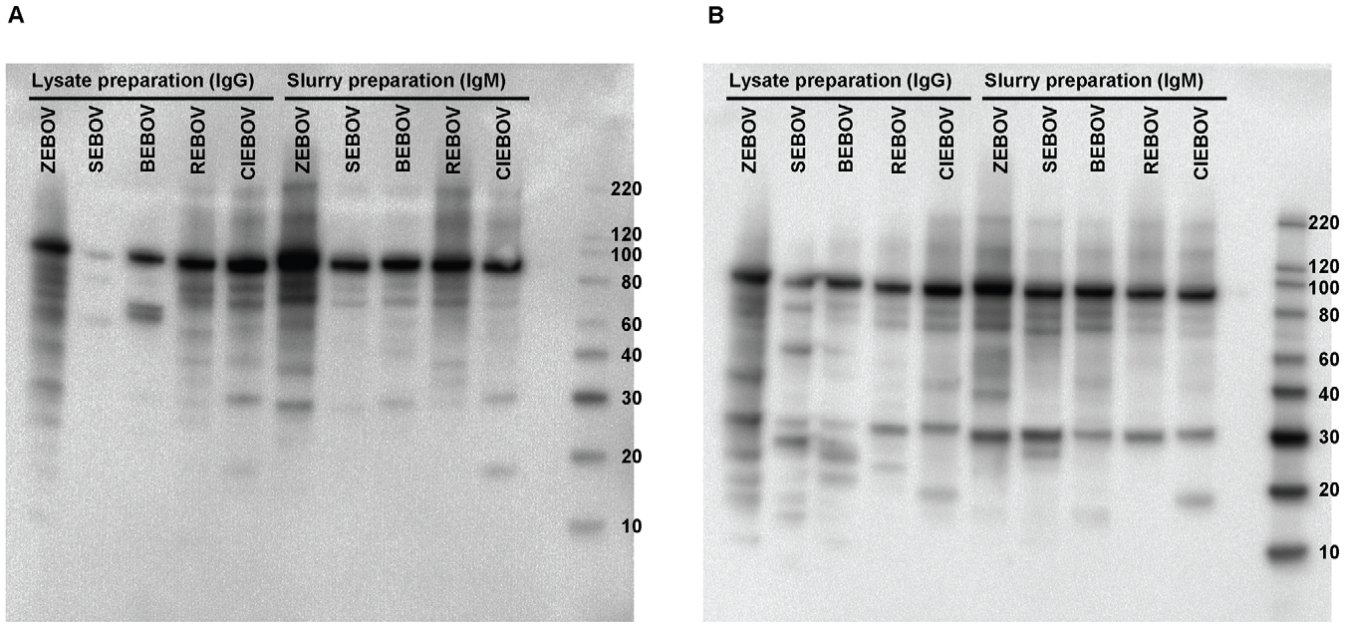
Western blots showing lysate and slurry antigen preparations for each EBOV species. HMAF poly-clonal antibodies against ZEBOV, SEBOV, REBOV, and CIEBOV (A) and rabbit poly-clonal antibodies against ZEBOV, SEBOV, and REBOV (B), were used as detector antibodies.

We produced non-recombinant infectious virus-based slurry (for IgM) and lysate (for IgG) antigen preparations for each of the five EBOV species. In order to confirm the presence of EBOV antigen in each viral lysate and slurry antigen preparations, we performed Western blots, using HMAF poly-clonal antibodies, raised against ZEBOV, SEBOV, REBOV, and CIEBOV (figure 1A) and rabbit poly-clonal antibodies, raised against ZEBOV, SEBOV, and REBOV (figure 1B), as detector antibodies. Although neither detector antibody mixture contained antibodies specifically raised against BEBOV, the presence of reactive nucleoprotein (NP) bands near the 100 kilodalton weight marker, both in the lysate and slurry preparations, indicates the presence of viral antigen in both of the preparations. While only a faint NP band was detected in the SEBOV lysate preparation using the HMAF detector antibody mixture, the presence of antigen was apparent using the rabbit polyclonal antibody mixture. This may suggest an issue in reactivity of the HMAF antibody mixture against the SEBOV lysate preparation. However, we noted the presence of a strong NP band in the SEBOV slurry preparation, using the HMAF antibody mixture, indicating the utility of the HMAF as a detector antibody for the SEBOV IgM ELISA assay.

**Figure 2:**
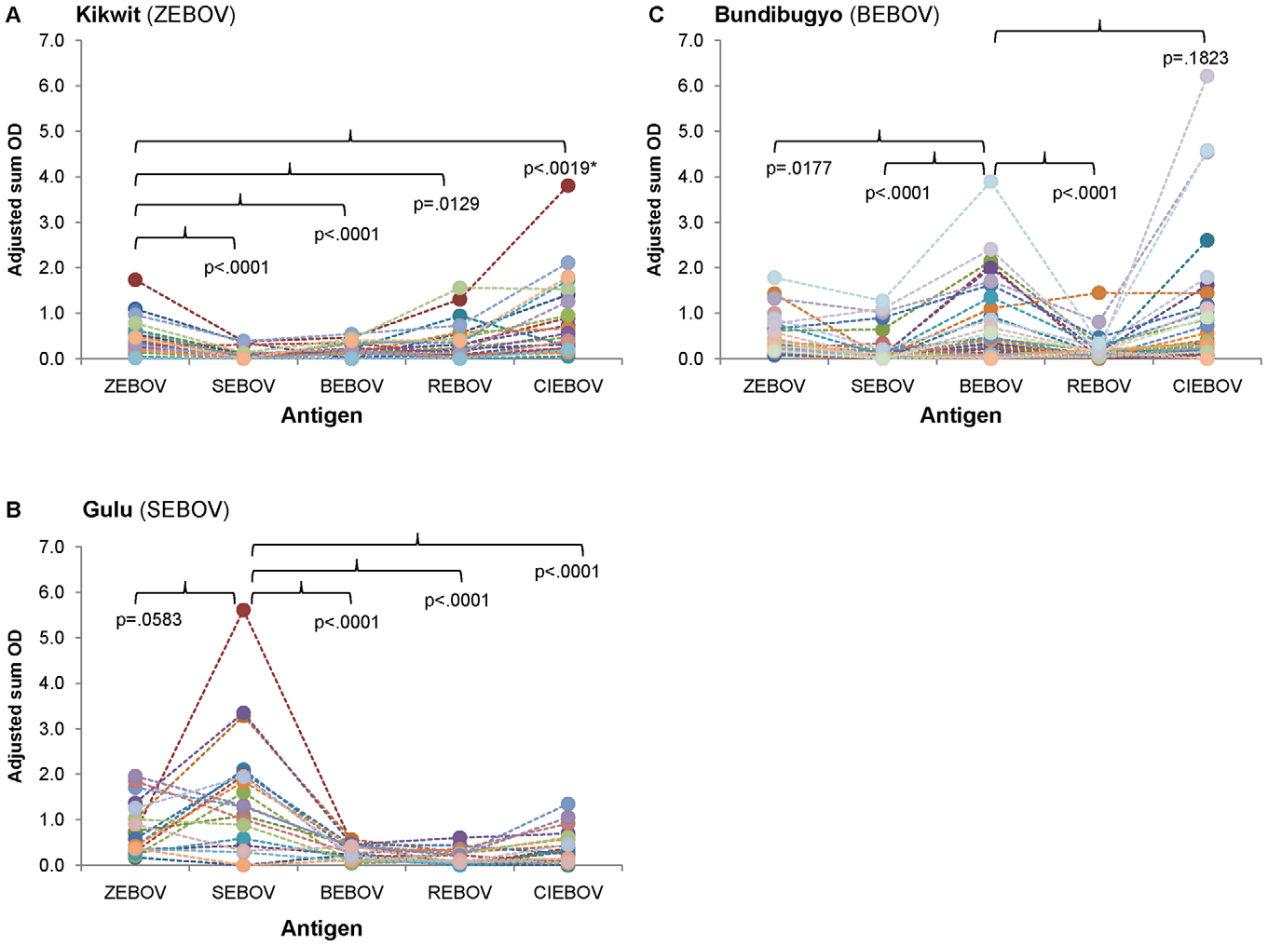
IgM adjusted sum OD values for outbreak samples to each of the five EBOV antigens. Panels represent samples from Kikwit (A), Gulu (B), and Bundibugyo (C). Closed circles correspond to the adjusted sum OD value for an individual sample to the specific EBOV antigen. Dotted lines are provided to notate the value of an individual sample across all antigens. Footnote: *Adjusted sum OD value is significantly higher to heterologous antigen than to autologous antigen.

Samples for this study are convalescent specimens collected as part of diagnostic activities for outbreaks due to ZEBOV (Kikwit, Democratic Republic of Congo, 1995 [5]), SEBOV (Gulu, Uganda, 2000 [19]), BEBOV (Bundibugyo, Uganda, 2007 [9]), and REBOV (Philippines [13]) (table 1). We quantitatively examined the IgM antibody reactivity to autologous versus heterologous virus antigen by comparing adjusted sum OD values for each of the individual virus slurry antigen preparations, among outbreak samples. While many of the samples, particularly from Kikwit and Bundibugyo, had low IgM titers, overall adjusted sum OD IgM values tended to be higher to autologous than heterologous virus antigen preparations (figure 2). For instance, adjusted sum OD values for samples from the Kikwit outbreak were significantly higher against ZEBOV antigen, than against SEBOV, BEBOV, and REBOV. Similar trends are also apparent for samples from the Gulu and Bundibugyo outbreaks. Interestingly we note that samples from the Kikwit outbreak had significantly higher adjusted sum OD values against CIEBOV than against ZEBOV, and additionally samples from Bundibugyo had higher (although not significantly different) values against CIEBOV than BEBOV antigen. All samples from the Philippines were demonstrated to be IgM negative during diagnostic testing and thus adjusted sum OD values were not examined in this study.

**Figure 3:**
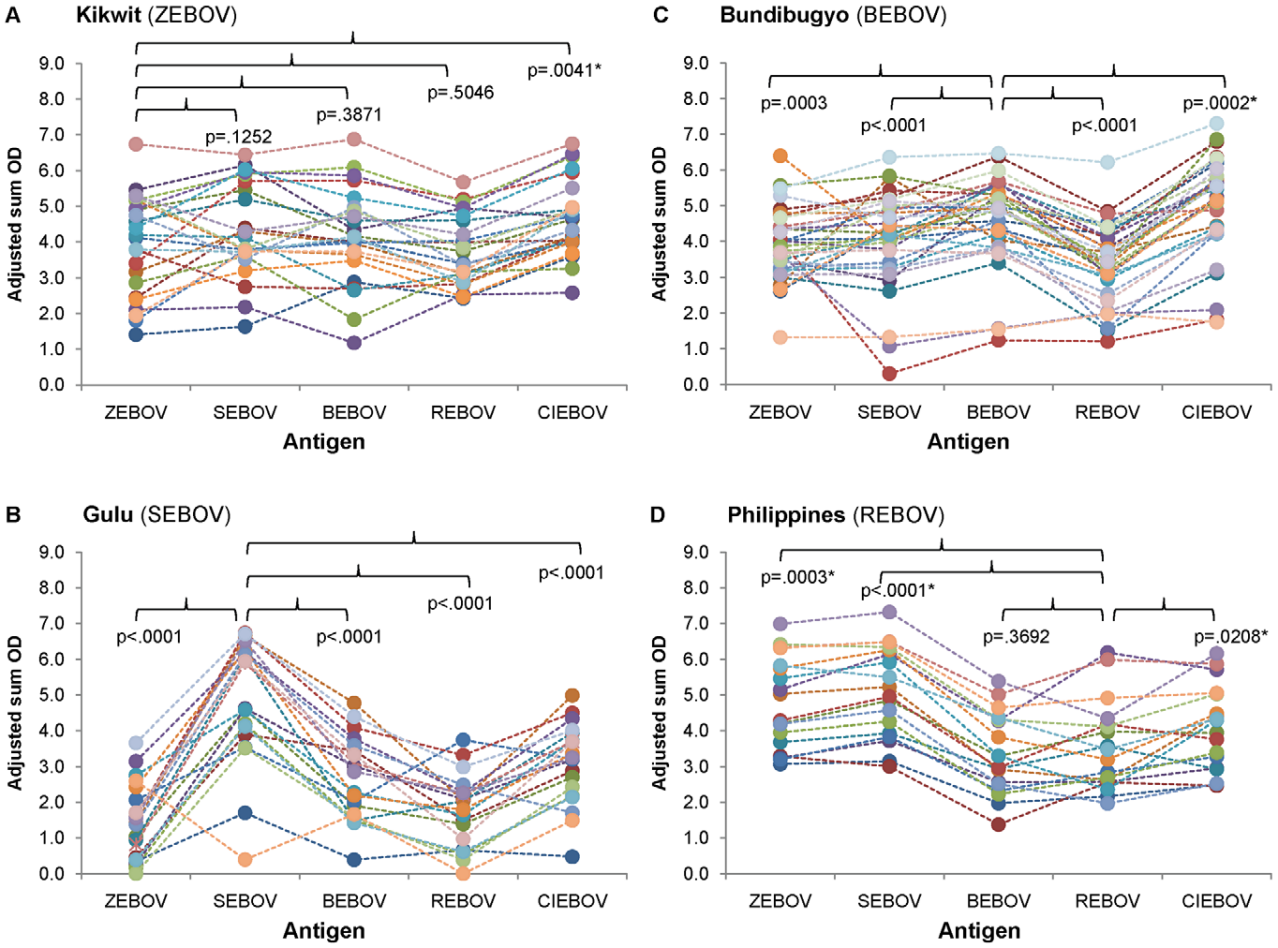
IgG adjusted sum OD values for outbreak samples to each of the five EBOV antigens. Panels represent samples from Kikwit (A), Gulu (B), Bundibugyo (C), and Philippines (D). Closed circles correspond to the adjusted sum OD value for an individual sample to the specific EBOV antigen. Dotted lines are provided to notate the value of an individual sample across all antigens. Footnote: *Adjusted sum OD value is significantly higher to heterologous antigen than to autologous antigen.

We additionally examined IgG antibody reactivity of autologous versus heterologous virus antigen by comparing adjusted sum OD values for each of the individual virus lysate antigen preparations among samples collected from each of the outbreaks. Owing to the convalescent stage at which most samples were collected, overall IgG adjusted sum OD values were mostly higher than IgM values (figure 3). Similar to trends observed for IgM responses, samples collected from Gulu and Bundibugyo outbreaks had significantly higher adjusted sum OD IgG values against autologous antigen than against heterologous antigen (with the exception of samples from Bundibugyo having higher values against CIEBOV than against BEBOV). In contrast, adjusted sum OD values for samples from Kikwit did not differ between ZEBOV and SEBOV, BEBOV, or REBOV, and had higher values for CIEBOV in comparison to ZEBOV antigen. Interestingly, samples from the Philippines had higher adjusted sum OD values against ZEBOV, SEBOV, and CIEBOV, than against autologous REBOV antigen.

**Figure 4:**
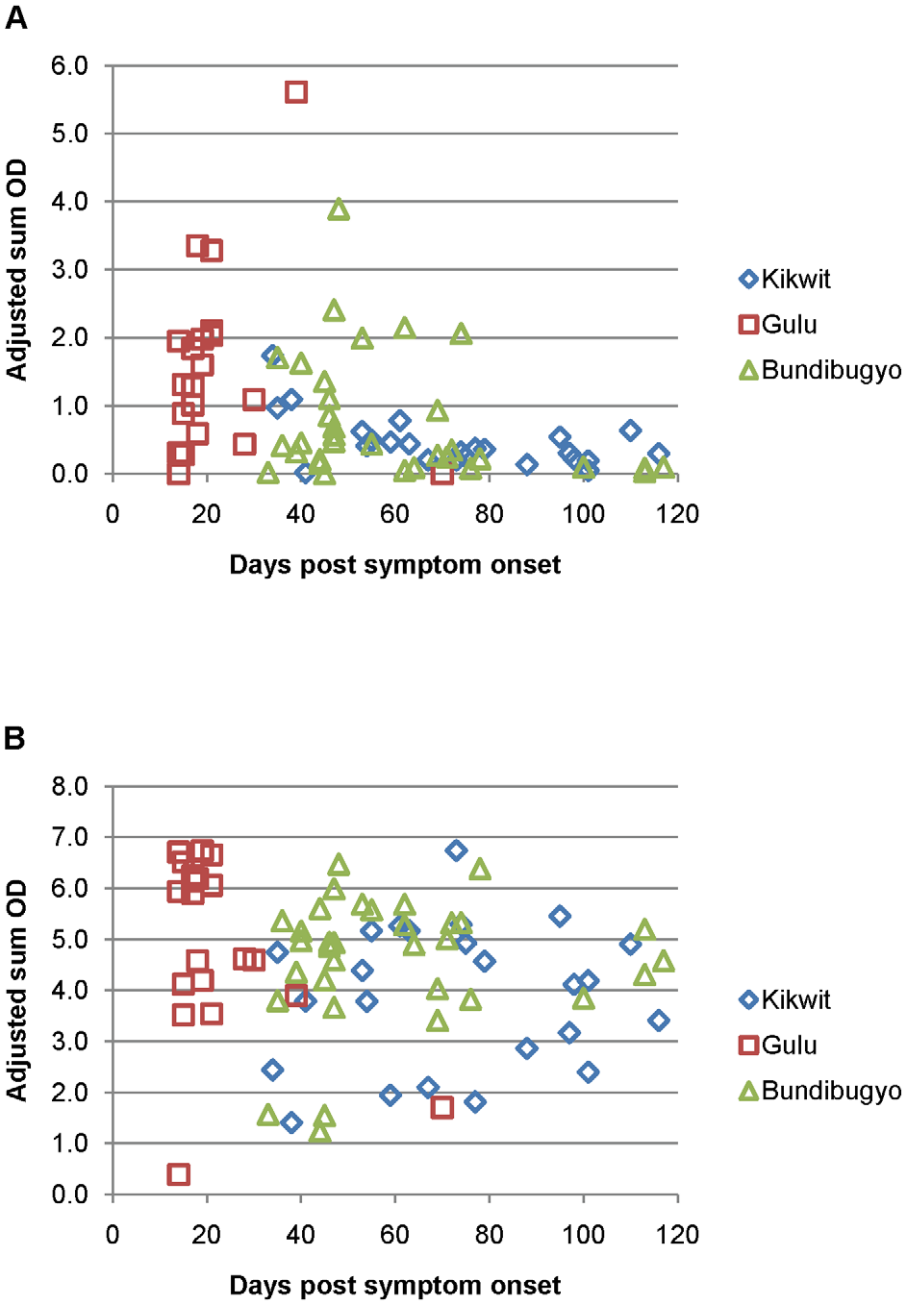
IgM and IgG adjusted sum OD values to autologous antigen by day of sample collection relative to symptom onset. Panels correspond to IgM (A) and IgG (B) assays.

We examined the kinetics of antibody development, for samples from Kikwit, Gulu, and Bundibugyo, by plotting the adjusted sum OD to autologous antigen for each of the sets of samples, relative to time post symptom onset (figure 4). The combined data for samples from these three outbreaks indicated early presence of IgM antibodies (earliest samples for this study were at 14 days post symptom onset). While sample collection dates varied for the Kikwit, Gulu, and Bundibugyo samples, adjusted sum OD values peaked between 30–50 days, and largely declined by 80 days post symptom onset. As with IgM, IgG antibodies were present, even in most early samples, however, adjusted sum OD values remained high over the full course (as long as 117 days) of sample collection post-symptom onset.

While the adjusted sum OD measure allowed us to quantitatively compare serologic cross-reactivity between autologous and heterologous antigens using a continous variable measure, we additionally wanted to examine the performance of heterologous antigen from a discrete (positive or negative) diagnostic standpoint. In order to assess the utility of heterologous antigen for the serologic diagnosis of EBOV infection by IgM ELISA, we selected all individuals with positive IgM antibody responses to respective autologous antigen from Kikwit, Gulu, and Bundibugyo outbreaks, and examined the sensitivity of the heterologous antigens for serologic diagnosis of EBOV in these samples (table 2). While the overall sensitivity of heterologous pairs varied widely, many heterologous virus combinations had low sensitivity for detection of positive IgM antibody responses. For instance, SEBOV, BEBOV, and REBOV antigen preparations had a sensitivity of less than 40% for all combinations of heterologous outbreak samples.

**Table 2:** Sensitivity of IgM ELISA, using heterologous antigen.

Outbreak (virus)	# Positive with autologous virus	# Positive, ZEBOV antigen (Sensitivity)	# Positive, SEBOV Antigen (Sensitivity)	# Positive, BEBOV Antigen (Sensitivity)	# Positive, REBOV Antigen (Sensitivity)	# Positive, CIEBOV Antigen (Sensitivity)
Kitwit (ZEBOV)	8		0 (0%)	1 (13%)	3 (38%)	6 (75%)
Gulu (SEBOV)	16	10 (67%)		2 (13%)	0 (0%)	7 (47%)
Bundibugyo (BEBOV)	15	8 (53%)	5 (34%)		2 (13%)	13 (87%)

We similarly examined the diagnostic utility of heterologous antigen for the serologic diagnosis of EBOV infection by IgG ELISA. In contrast to the above results for the IgM assay, heterologous antigens had a high sensitivity in the detection of IgG antibodies (table 3). With the exception of samples from Gulu, which displayed a diagnostic sensitivity of 74% with ZEBOV and REBOV antigen, all heterologous antigen pairs displayed at least 95% sensitivity for detection of IgG antibodies, and for many combinations, heterologous antigen detected positive results for 100% of samples.

**Table 3:** Sensitivity of IgG ELISA, using heterologous antigen.

Outbreak (virus)	# Positive with autologous virus	# Positive, ZEBOV antigen (Sensitivity)	# Positive, SEBOV Antigen (Sensitivity)	# Positive, BEBOV Antigen (Sensitivity)	# Positive, REBOV Antigen (Sensitivity)	# Positive, CIEBOV Antigen (Sensitivity)
Kitwit (ZEBOV)	24		24 (100%)	24 (100%)	24 (100%)	24 (100%)
Gulu (SEBOV)	19	14 (74%)		18 (95%)	14 (74%)	18 (95%)
Bundibugyo (BEBOV)	33	33 (100%)	33 (100%)		33 (100%)	33 (100%)
Philippines (REBOV)	18	18 (100%)	18 (100%)	18 (100%)		18 (100%)

## Discussion

The precise nature of antibody cross-reactivity between EBOV species has not been fully characterized. Some studies have reported detectable antibody reactivity to heterologous antigen in serum from humans or animals [25], [27], [30], [31], [32], as well as noted potential differences in the cross-reactivity between autologous and heterologous antigen [27], [32], [33]. However, interpretation of these results remains difficult, owing the differences in antigen (whole virus versus recombinant antigen) and overall sample size, for many previous studies. In this study, among samples from the Kikwit, Gulu, and Bundibugyo outbreaks, we consistently observed higher adjusted sum OD values for IgM antibody responses against autologous antigen than against heterologous antigens. While IgM antibody responses were low for many samples (in contrast to IgG responses), when we limited our analysis to those samples that were positive to autologous antigen on the basis of diagnostic IgM criteria, we observed low sensitivity of the IgM ELISA to heterologous antigen. Although some samples did react to heterologous antigen, our data indicate a species-specificity of IgM antibody responses in individuals infected with EBOV.

In contrast to IgM antibody responses, IgG antibodies consistently displayed cross-reactivity to heterologous antigen, as demonstrated by the high adjusted sum OD values to heterologous antigens, as well as the high sensitivity of the IgG ELISA as a diagnostic assay. Previous serosurveys in Gabon, Central Africa Republic, and Democratic Republic of Congo have reported prevalence of anti-EBOV antibodies in rural populations ranging from (5–15%), which were presumed as indicative of previous infection with ZEBOV [34], [35], [36]. Owing to the high degree of IgG cross-reactivity we observed in this study, it is possible the relatively high seroprevalence of anti-EBOV antibodies reported in these studies may be the result of exposure to an unknown EBOV species, with lower pathogen potential than ZEBOV.

The kinetics of antibody response to EBOV in humans has been best described for ZEBOV. Ksiazek et al reported early onset and peaking (∼18 days) of IgM responses, which largely diminished by 60 days post-infection, while IgG antibodies were also present early post-onset and persisted for months following infection, in survivors [17]. Similar observations were reported by Baize et al. [18] and recent data from Wauquier et al. indicated that ZEBOV-reactive IgG antibodies persist for years following EHF [37]. While the samples examined in this study are not uniform with regard to time post-symptom onset, relative the EHF outbreak, our data do suggest the above observations can be extended for other EBOV species.

An unexpected finding in this study was the overall high level of seroreactivity of heterologous samples to CIEBOV antigen. For instance, IgM adjusted sum OD values for samples from Kikwit, and IgG adjusted sum OD values from Kikwit, Bundibugyo, and the Philippines, were all significantly higher for heterologous CIEBOV antigen than for autologous antigen. The reason for this observation is unclear, however these are likely not the result of higher concentrations of CIEBOV antigen in lysate and slurry preparations, as demonstrated by the similar antigen concentration of CIEBOV antigen to the other antigen preparations in Western blot. It would be of interest to compare the cross-reactivity of human anti-CIEBOV sera, between autologous and heterologous EBOV antigen, however because of the scarcity of identified human infections (only one patient diagnosed) with to CIEBOV, we were unable to address this question.

In addition, for samples from the Philippines, adjusted sum OD values for IgG tended to be higher against heterologous antigens than again REBOV antigen. We do not have an explanation for this observation, however, owing to the absence of detectable IgM responses in any samples from the Philippines, and the apparent lack of symptomatic disease in humans exposed to REBOV, these samples could represent later stage serologic responses in comparison to the other groups of samples, and may potentially include individuals with boosted immune responses due to multiple previous exposures to REBOV.

Our observations indicate limitations in the utility of IgM ELISA, for diagnosis of EHF, prior to identification of the virus species. However, previous studies have reported early development of IgG antibodies in surviving EHF cases [17], [18] (and similarly supported by temporal data from this study). Because the IgG ELISA detected positive IgG antibody responses for the majority of samples with heterologous antigen, IgG ELISA assays in late-acute or early-convalescent samples may effectively circumvent the limitations in IgM ELISA, for diagnosis of EHF when the viral species is not known.

We do note limitations of our study. The limited availability of diagnostic sera prohibited the opportunity to examine antibody cross-reactivity to specific EBOV proteins, or to specific epitopes. While the antigenic preparations used for ELISA in our study may be modestly enriched for NP, this approach does not preclude other proteins (as demonstrated by Western blot in figure 1) and antigen preparations were used at high concentrations for the ELISAs [17], [27]. For instance, in a recent study, Becquart et al. used the same IgG ELISA (including ZEBOV antigen produced in the same manner) as this current study to identify a large number of seropositive individuals, and confirmed EBOV-specific antibody responses in 138 individuals by Western blot. All individuals reacted to at least one viral protein, however, only 56% displayed antibody reactivity to NP [36].

Secondly, while we quantified IgM and IgG antibody levels, these do not necessarily represent the presence or quantity of neutralizing antibodies. The kinetics of development and functional role of neutralizing antibodies in viral clearance and protection in humans in not well understood. Currently most known neutralizing antibodies to EBOV target epitopes in the viral glycoprotein (GP), and data suggest GP as an important protein for viral neutralization [38], [39], [40], [41], [42]. Interestingly, in studies involving humans with evidence of asymptomatic infection [22] and humans seropositive to EBOV [36], the most common seroreactive proteins by Western blot were VP40 and NP; only a minority of individuals displayed evidence of reactive antibodies to GP. Although it is possible that antibody responses have a limited role in protective immunity to EBOV in humans, data from these studies (living individuals with evidence of previous EBOV infection) as well as from outbreak studies [17], [18], support the notion that antibody responses are an important correlate of immunity to EBOV in humans.

In summary, we assessed the cross-reactive nature of IgM and IgG antibodies from groups of human survivors who were infected with four different species of EBOV. We observed cross-reactivity of IgG antibodies to heterologous antigen, however, overall reactivity to IgM and IgG antibodies tended to be stronger for autologous than heterologous antigen. Some experimental vaccines have suggested limited cross-protection of heterologous EBOV antigen [43]. Hensley et al. recently reported cross-protection against BEBOV infection in cynomolgus macaques vaccinated with DNA/rAd5 vaccine expressing GP of ZEBOV and SEBOV, although concluded that protection was the result of cellular immunity [33]. Our data suggest potential utility of heterologous vaccine for protection against EBOV, should IgG antibody responses prove to be an effective mediator of immunity to EBOV in humans.
